# Association of hippocampus, entorhinal cortex, and amygdala with thyroid function: a bilateral volumetric analysis

**DOI:** 10.1186/s13044-026-00289-4

**Published:** 2026-02-23

**Authors:** Asma Hallab

**Affiliations:** 1 Psychiatry Neuroimaging Laboratory - Psychiatry and Radiology Departments – Mass General Brigham, Harvard Medical School, Boston, MA USA; 2 Biologie Intégrative et Physiologie – Neurosciences Cellulaires et Intégrées, Faculté des Sciences et Ingénierie, Sorbonne Université, Paris, France; 3 Charité - Universitätsmedizin Berlin, Corporate Member of Freie Universität Berlin and Humboldt-Universität zu Berlin, Charitéplatz 1, 10117 Berlin, Germany

**Keywords:** Brain, Neurodegeneration, Psychoneuroendocrinology, Thyroid stimulating hormone, Magnetic resonance imaging, Cognitive neurosciences

## Abstract

Thyroid hormones modulate brain structures during neurogenesis and impact cognition and emotions during the lifetime. Hypothyroidism is commonly associated with lower gray matter volume. Fewer studies, however, highlighted significant associations between higher thyroid function and cognitive impairment. Aging is an independent risk factor for cognitive decline and thyroid dysfunction. It is, therefore, important to understand the association of thyroid function with relevant brain structures during the aging process. A subset of 1,010 adults (≥ 50 years) from the Alzheimer’s Disease Neuroimaging Initiative (ADNI) was included. Multivariable linear regression was used. Comparative bilateral volumetric analyses and interaction-based cognition-related stratifications were performed. Females represented 50.00% of included cases, and 43.50% were healthy controls (HC). Thyroid Stimulating Hormone (TSH) levels were lower in the mild cognitive impairment (MCI) group (1.67 vs. 1.81 µIU/mL, *p*-value = 0.024). A significant positive association between TSH (µIU/mL) and left hippocampus volume (mm^3^) was found in MCI (adj.*ß*=44.00 [7.50, 81.00], *p-value* = 0.018). Similar results were found in the right hippocampus, but only in the total study population (adj.*ß*=29.00 [4.00, 54.00], *p-value* = 0.023). There was a significant association between TSH (µIU/mL) and left EC volume (mm^3^) in the total study population (adj.*ß*=31.00 [9.40, 52.00], *p-value* = 0.005), and after stratification, this association remained statistically significant only in MCI (adj.*ß*=54.00 [24.00, 84.00], *p-value* < 0.001). The association between TSH and the amygdala was not statistically significant. It is, therefore, crucial to consider the role of cognitive status and laterality when exploring the thyroid-brain interaction in older adults.

**Supplementary Information:**

The online version contains supplementary material available at 10.1186/s13044-026-00289-4.

## Clinical trial number

Not applicable.

**Supplementary Information:**

The online version contains supplementary material available at 10.1186/s13044-026-00289-4.

## Highlights

Lower TSH levels were associated with lower left hippocampus volume in MCI.

Lower TSH levels were associated with lower left entorhinal cortex volume in MCI.

No significant association between TSH and bilateral amygdala volumes was observed.

**Supplementary Information:**

The online version contains supplementary material available at 10.1186/s13044-026-00289-4.

## Graphical Abstract

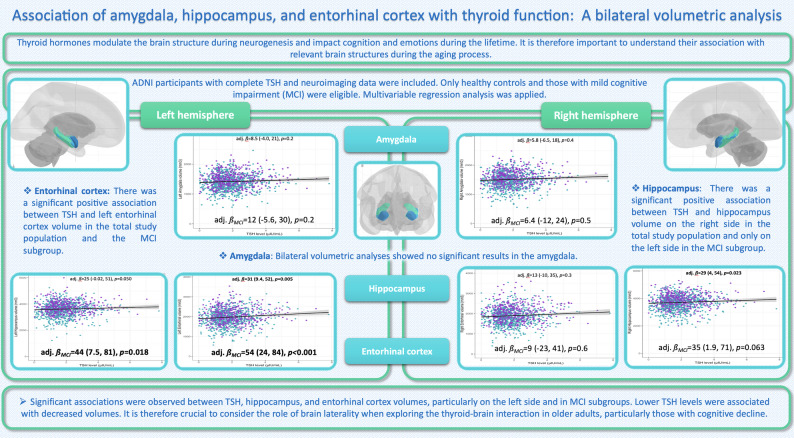

## Introduction

The thyroid-brain association is a multifaceted interaction that, despite starting very early during gestational neurogenesis, remains relevant during adulthood and the aging process, making it of interest to all age spans and medical disciplines [1]. Hypothyroidism, commonly described in older adults, is associated with lower gray matter volume [2, 3]. Physiologically, thyroid function slowly declines during aging, resulting in normally increased Thyroid-Stimulating Hormone (TSH) levels in older adults [4, 5]. However, aging is also a risk factor for thyroid dysfunction and pathology [5]. Around 4% of older adults are diagnosed with hypothyroidism, with a higher prevalence among females [6]. The overall prevalence of thyroid dysfunction in older adults might reach 24% [7], while the prevalence is significantly lower in young and middle-aged adults (age-standardized prevalence around 5% in a representative American population) [8]. Similarly, aging is an independent risk factor for cognitive decline [9]. It is expected that 153 million of the world’s population will develop some form of dementia by 2050 [10]. The association between thyroid function and cognitive decline in advanced ages has motivated several studies and raised various controversies, depending on the studied populations, clinical definitions of thyroid (dys)function, and psychometric variables [11, 12]. While those studies put a particular focus on the association between hypothyroidism and cognitive decline, particularly in older adults, a smaller number of publications highlighted the association between lower TSH levels and cognitive impairment in the same age group [13, 14]. Our previous studies based on the Alzheimer’s Disease Neuroimaging Initiative (ADNI) population showed a significant association between lower TSH levels, cognitive impairment [15], and perceived symptoms of anxiety in 50-year-old and older adults [16]. 

It is, therefore, crucial to understand the underlying mechanisms defining the association between thyroid function and neuropsychiatric symptoms, particularly in older adults, a high-risk group of neurocognitive and neuropsychiatric adversities [17, 18]. Structural brain analysis is a relevant tool for understanding and quantifying volume loss and neurodegeneration [19, 20]. Limbic structures in the medial temporal lobe (MTL), mainly the hippocampus and entorhinal cortex (EC), are particularly relevant centers for cognition and are early affected during the neurodegenerative process and dementia [21, 22]. Furthermore, the amygdala, another limbic structure of the MTL, is associated with memory formation [22] and emotional processing, and plays a central role in experiencing and expressing survival-relevant emotions such as fear and anxiety [23]. It is also known that brain laterality has several implications for psychomotor and cognitive outcomes, and diverse factors, such as aging, might influence its structural and functional organization [24].

Despite the largely discussed clinical evidence in favor of an interplay between thyroid and cognition across different age groups, very limited data are available on the association between thyroid function and brain structures in older adults. It is also unclear how the neurocognitive status might modulate this association, and whether brain function laterality might reflect structural discrepancies in the thyroid-brain interaction.

Therefore, the aim of the current study was (A) to study the overall association between TSH and the volumes of the hippocampus, EC, and amygdala while highlighting the relevance of a bilateral volumetric approach; and (B) to evaluate the value of cognitive status-related stratification in this association.

## Methods

The study was conceptualized and reported according to STrengthening the Reporting of OBservational studies in Epidemiology (STROBE) guidelines [25]. 

### Study population

ADNI is a non-interventional longitudinal cohort initiated by the principal investigator, Dr. Michael Weiner, and funded by the National Institute on Aging (National Institutes of Health Grant U19 AG024904). The main objective of this cohort was to understand dementia and related risks. Healthy adults aged 50 years and older, as well as those with cognitive impairment, were eligible. The recruitment of study participants took place in several centers across the United States of America and Canada. Neurocognitive, neuroimaging, and biological biomarkers were collected during recurrent study visits. Participants gave written informed consent. The study was performed according to the Declaration of Helsinki, and ethical approvals were obtained from each ADNI’s local recruitment center’s IRB. Details and protocols can be found at https://adni.loni.usc.edu.

### Thyroid function

TSH is a relevant biomarker of central thyroid function and was measured in fasting blood at baseline and reported in µIU/mL. Complete and accurate TSH measurements were individually screened, and only the first value was retained in case of duplicate measurements. Very low indetectable values (< 0.01 µIU/mL) were converted to 0.01 µIU/mL in three cases. Cases with TSH values equal to or higher than 10 µIU/mL (higher probability of overt hypothyroidism) were excluded from the analysis.

### Neuroimaging and brain segmentations

ADNI participants underwent cerebral 1.5 or 3 Tesla magnetic resonance imaging (MRI) scans at baseline, depending on the study phase (ADNI 1 versus ADNI go, 2, 3). Owing to variations in protocols and techniques used in each ADNI phase and recruitment center, details exceed the frame of this work, and protocols are published at https://adni.loni.usc.edu/data-samples/adni-data/neuroimaging/mri/. Several manuscripts described technical details of neuroimaging methods applied in different ADNI phases [26, 27]. For the current study, volumes of interest (VOIs) were selected based on a rigorous review of the literature and previous findings on the same population [15, 16]. The volumetric analysis was performed with T_1_-based segmentations using FreeSurfer (https://surfer.nmr.mgh.harvard.edu). The left and right hippocampus, left and right EC, and left and right amygdala were assessed. The total intracranial volume (ICV) was also reported and introduced to the multivariable regression models to adjust for anatomical variations (mm^3^). Owing to the variation in MRI scanners (non-accelerated 1.5 Tesla, accelerated 1.5 Tesla, and 3 Tesla) used, the type of scanner was also adjusted for. All volumes are measured in mm.

### Neuropsychological assessments

The cognitive status was mainly assessed using the total score of the Alzheimer’s Disease Assessment Scale – Cognitive subscale 13 items (ADAS_13_) [28], in addition to total scores of the Clinical Dementia Rating scale – Sum of Boxes (CDR-SB) [29], and Functional Activities Questionnaire (FAQ) [30]. Depression symptoms were assessed using the total score of the Geriatric Depression Scale (GDS) [31]. Anxiety symptoms were reported by study partners in the Neuropsychiatry Inventory Questionnaire (NPI-Q) [32]. 

### Inclusion criteria

Only participants with mild cognitive impairment (MCI) and healthy controls (HC) were eligible. Variables were missing in TSH measurements (*n* = 159), relevant VOIs (*n* = 907 hippocampus, *n* = 911 EC, and *n* = 910 amygdala), main diagnosis at baseline (*n* = 21), demographical information (*n* = 4 missing age), ADAS_13_ total score (*n* = 25), GDS total score (*n* = 3), biometric information for the BMI (*n* = 6), and APOE ε4 status (*n* = 217). Probably erroneous weight or height measurements (*n* = 2), TSH ≥ 10 µIU/mL (*n* = 2), time between TSH measurement and MRI scan exceeding 60 days (*n* = 38), and cases with dementia (*n* = 413) were also excluded. This allowed the inclusion of 1,010 cases with complete data.

### Statistical analyses

RStudio version 2024.12.1–563 was applied for the analyses and visualization of the data. Values were presented as medians with interquartile ranges (IQR) or numbers with percentages (%) in continuous or count data. The associations between VOI volumes and TSH levels were assessed using linear regression models, where the VOI volume (mm^3^), as a continuous value, was the predicted variable, and continuous TSH levels (µIU/mL) as the predicting variable. A crude model was initially presented, and then the model was adjusted for relevant confounders: Age (years), sex, ADAS_13_ total score (points), educational level (years), APOE ε4 status (“None”, “one”, or “two alleles”), cognition-related main diagnosis (“HC” and “MCI”), ICV (mm^3^), type of MRI scanner (“non-accelerated 1.5 Tesla”, “accelerated 1.5 Tesla”, and “3 Tesla”), time between TSH measurement and MRI scan (days), and GDS total score (points).

An interaction term was introduced to the model (TSH*Cognition-related main diagnosis), and when statistically significant, a diagnosis-based stratification was performed. The same strategy of model adjustment was followed after stratification, and adjusted models were adapted correspondingly by removing the predictor “main diagnosis”. The analyses were performed with VOIs corresponding to the bilateral structures, each independently. Results were presented as regression coefficient (*ß*), 95% confidence interval (*CI*), and the *p*-value. The significance level of the two-sided *p*-value was set at 0.050 (significant *p-value* < 0.050). For the anatomical structures, no correction for multiple testing was needed, as the current VOI analyses were based on prior hypotheses driven by clinical studies [3, 15, 16, 33, 34], as well as rodent studies [35–37], and no random search for statistical significance of anatomical structures was followed during this work.

## Results

### Description of the study population

From the total 1,010 population, 439 (43.50%) were HC and 571 (56.50%) were MCI cases. Females represented 50% of the total population (*n* = 508). The median serum TSH level was 1.76 µIU/mL (IQR: 1.19–2.45). Detailed information on the study population and cognition-related subgroups is summarized in Table 1.

**Table 1:** Characteristics of the study population and group comparison

Characteristics	Overall, *N* = 1,010 (100%)^1^	Healthy controls *N* = 439 (43.5%)^1^	Mild cognitive impairment *N* = 571 (56.5%)^1^	*p*-value^2^
**Age (years)**	71 (67, 77)	71 (67, 76)	72 (66, 77)	0.500
**Sex (Female)**	508 (50%)	259 (59%)	249 (44%)	**< 0.001**
**Ethnicity**				**0.047**
White	915 (91%)	387 (88%)	528 (92%)	
Black	53 (5%)	31 (7%)	22 (4%)	
Other	42 (4%)	21 (5%)	21 (4%)	
**Marital status**				0.130
Currently married	753 (75%)	317 (72%)	436 (76%)	
Not married or unknown	257 (25%)	122 (28%)	135 (24%)	
**Housing situation**				0.600
House or appartment	957 (95%)	414 (94%)	543 (95%)	
Nursing institution	35 (3%)	18 (4%)	17 (3%)	
Other	18 (2%)	7 (2%)	11 (2%)	
**APOE ε4**				**< 0.001**
No alleles	605 (60%)	301 (69%)	304 (53%)	
1 allele	333 (33%)	122 (28%)	211 (37%)	
2 alleles	72 (7%)	16 (3%)	56 (10%)	
**ADAS**_**13**_ **(points)**	11 (7, 16)	8 (5, 11)	14 (10, 19)	**< 0.001**
**FAQ total score (points)**	0 (0, 2)	0 (0, 0)	1 (0, 4)	**< 0.001**
Missing value	6	2	4	
**CDR-SB (points)**	0.50 (0.00, 1.50)	0.00 (0.00, 0.00)	1.00 (1.00, 2.00)	**< 0.001**
**GDS total score (points)**	1 (0, 2)	0 (0, 1)	1 (1, 3)	**< 0.001**
**BMI**	26.7 (24.40, 30.10)	26.7 (24.30, 30.00)	26.7 (24.50, 30.10)	0.500
**TSH (µIU/mL)**	1.76 (1.19, 2.45)	1.81 (1.23, 2.56)	1.67 (1.15, 2.37)	**0.024**
**Left hippocampus (mm** ^**3**^ **)**	3,630 (3,304, 3,963)	3,748 (3,449, 4,040)	3,535 (3,165, 3,871)	**< 0.001**
**Right hippocampus (mm** ^**3**^ **)**	3,743 (3,380, 4,062)	3,856 (3,554, 4,151)	3,643 (3,227, 3,988)	**< 0.001**
**Left entorhinal cortex (mm** ^**3**^ **)**	1,962 (1,730, 2,223)	2,006 (1,831, 2,260)	1,927 (1,663, 2,188)	**< 0.001**
**Left entorhinal cortex (mm** ^**3**^ **)**	1,872 (1,588, 2,150)	1,970 (1,715, 2,234)	1,800 (1,505, 2,057)	**< 0.001**
**Left amygdala (mm** ^**3**^ **)**	1,409 (1,238, 1,577)	1,463 (1,320, 1,608)	1,366 (1,200, 1,539)	**< 0.001**
**Right amygdala (mm** ^**3**^ **)**	1,500 (1,340, 1,670)	1,551 (1,410, 1,718)	1,450 (1,268, 1,623)	**< 0.001**
**MRI-Scanner**				**< 0.001**
3 Tesla	358 (35%)	236 (54%)	122 (21%)	
Accelerated 1.5 Tesla	113 (11%)	26 (6%)	87 (15%)	
Non-Accelerated 1.5 Tesla	539 (53%)	177 (40%)	362 (63%)	

^1^ Median (IQR); n (%), ^2^ Wilcoxon rank sum test; Pearson’s Chi-squared test

ADAS_13_: Alzheimer’s Disease Assessment Scale – 13 items, APOE: Apolipoprotein, BMI: Body Mass Index, CDR-SB: Clinical Dementia Rating-Sum of Boxes,GDS: Geriatric Depression Scale, MRI: Magnetic Resonance Imaging, TSH: Thyroid Stimulating Hormone

### Hippocampus and central thyroid function

#### Left hippocampus

The association between serum TSH levels (µIU/mL) and the left hippocampus volume (mm^3^) in the total study population missed slightly the significance level (adj. *ß*_*Left hippocampus−Total population*_=25.00 [-0.02, 51.00], *p-value* = 0.050). The introduction of the interaction term between TSH and cognition-specific diagnosis showed significant results (TSH*MCI: adj. *ß*_*Left hippocampus*_=50.00 [0.50, 100.00], *p*-value = 0.048). After stratification, significant associations were observed in cases with MCI (adj. *ß*_*Left hippocampus−MCI*_=44.00 [7.50, 81.00], *p-value* = 0.018).

#### Right hippocampus

The association between serum TSH levels (µIU/mL) and the right hippocampus (mm^3^) was statistically significant: adj. *ß*_*Right hippocampus−Total population*_=29.00 [4.00, 54.00], *p-value* = 0.023. The introduction of the interaction term between TSH and cognition-specific diagnosis did not show significant results (*p*-value = 0.400). For the comparability of the findings with the left side, a stratification was visualized. The association in the MCI subpopulation missed slightly the significance level.

**Fig. 1:**
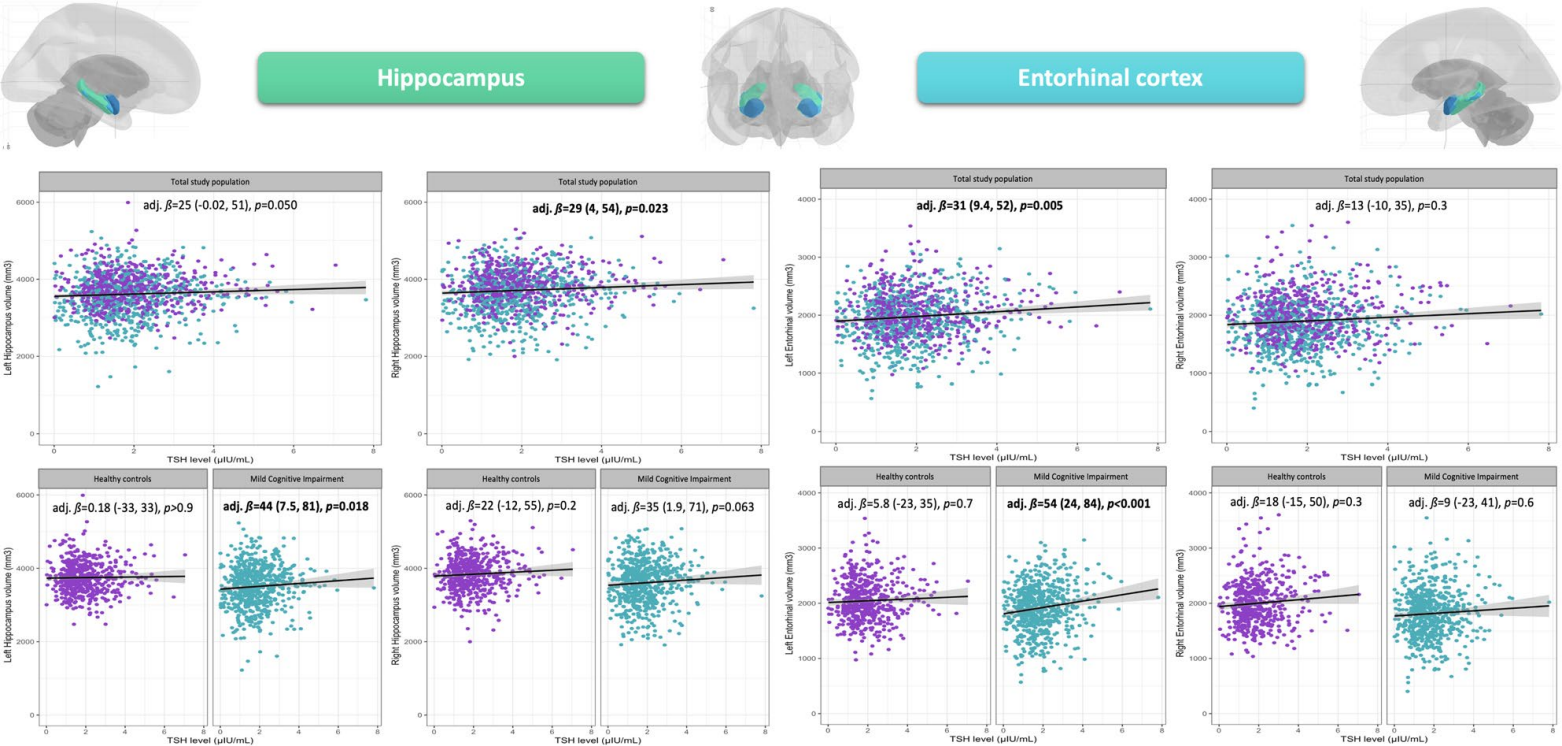
Adjusted linear regression models on the association between TSH levels, *left and right hippocampus*,* and left and right entorhinal cortex volumes*

Associations between TSH (µIU/mL) and the hippocampus volume (mm^3^) are visualized and summarized in Fig. 1 and detailed in Supplementary Table 1.

### Entorhinal cortex and central thyroid function

#### Left entorhinal cortex

There was a significant association between serum TSH levels and the Left EC volume (mm^3^) in the total study population (adj. *ß*_*Left EC−Total population*_=31.00 [9.40, 52.00], *p-value* = 0.005). The introduction of the interaction term between TSH and cognition-specific diagnoses showed significant results (TSH*MCI: adj. *ß*_*Left entorhinal cortex*_=48.00 [5.50, 90.00], *p*-value = 0.027). The stratification showed a stronger significant association between TSH levels and the left EC volume (mm^3^) only in MCI participants (adj. *ß*_*Left EC−MCI*_=54.00 [24.00, 84.00], *p-value* < 0.001).

#### Right entorhinal cortex

No significant associations were found in the right EC.

Associations between TSH and EC are visualized in Fig. 1 and detailed in Supplementary Table 2.

### Amygdala and central thyroid function

**Fig. 2:**
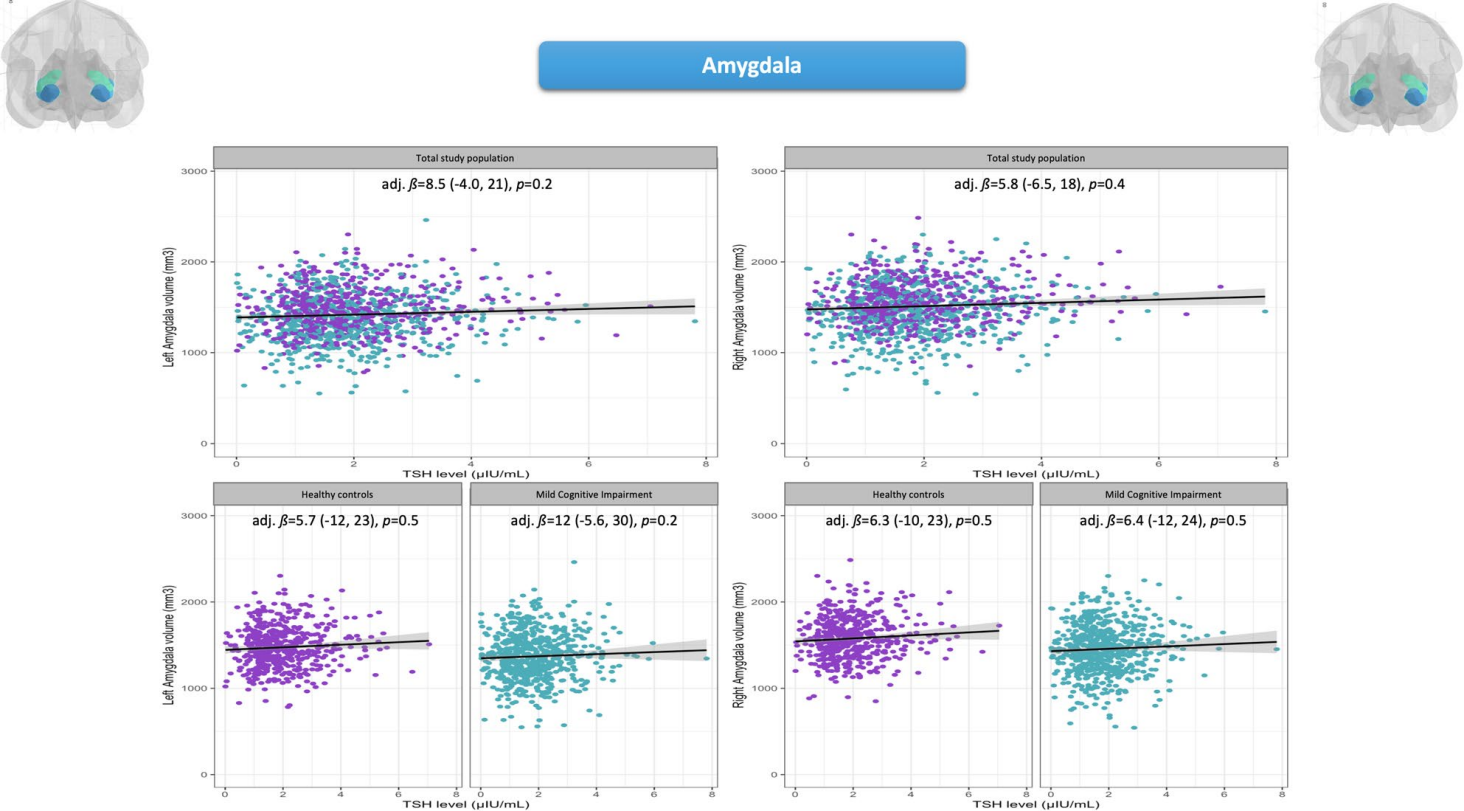
Adjusted linear regression models on the association between TSH levels and *left and right amygdala volume*

There was no significant association between serum TSH levels and the bilateral amygdala volumes, neither in the total study population nor after stratification, applied for comparability with other regions. Details are visualized in Fig. 2 and detailed in Supplementary Table 3.

## Discussion

The study explored the association between TSH levels and the volumes of three major limbic structures in middle-aged and older adults. The main outcome was the significant association between lower TSH levels, hippocampus, and EC volumes, depending on the studied side (particularly left) and cognition-related stratum (MCI). The bilateral VOI analyses highlighted the importance of exploring this association in left and right brain structures independently.

### Thyroid and hippocampus

A very limited number of published studies evaluated the association between thyroid function and hippocampus volume, particularly in older adults. In a study including 62 premenopausal women with newly diagnosed Graves’ disease and hyperthyroidism, volumes of both hippocampi were significantly smaller compared to healthy controls [34]. After treatment and correction of the hyperthyroidism, hippocampus volumes (right and left) increased significantly in size. Patients with normal and pathological TSH levels did not show significant differences in hippocampus volumes during the follow-up [34]. On a longitudinal course, older adults with Alzheimer’s dementia, but not healthy or MCI controls, showed a significant association between lower TSH levels and annual hippocampal volume loss, particularly on the left side [38]. In the same study, in contrast with the longitudinal analysis, the association between TSH and bilateral hippocampus volumes was not statistically significant in their baseline cross-sectional analysis [38]. Similar findings in the hippocampus were reported by another study, where cases with untreated hyperthyroidism had significantly lower bilateral hippocampus volumes compared to healthy controls [39]. The association between hyperthyroidism and diffuse gray matter atrophy, rather than regional, was described in a further study, where specifically hypothalamic subfields and limbic structures were explored, but showed no significant differences between cases and controls [40]. 

From a different TSH range- and age-related perspective, adolescents with a history of congenital hypothyroidism showed smaller hippocampal volumes, particularly on the left side, compared to healthy controls. Furthermore, they neither showed a proportionate increase in hippocampal size with age compared to the control group, nor a typical lateralization of cognitive functions [41]. In another study, newly diagnosed adults with subclinical or overt hypothyroidism presented lower hippocampal subfield volumes, particularly on the right side, compared to HC [33]. Similarly, lower right hippocampus volumes were observed in adults with hypothyroidism compared to those without [3]. In a large population study, higher TSH levels were associated with lower total brain volumes, mainly attributed to lower white matter than gray matter volume [42]. Further brain regions, such as the pallidum, also showed a significant association between neurodegeneration and hypothyroidism [2]. Furthermore, adults newly-diagnosed with hypothyroidism showed reduced gray matter volume in the fronto-temporal regions [43], but not in limbic structures. In a different population of antipsychotic-naïve patients with first-episode psychosis, high TSH levels were significantly correlated with lower total hippocampus volume at baseline [44]. 

The results of published data are in favor of lower hippocampus volume in hypothyroidism and hyperthyroidism. In our study, cases with dementia were excluded since their advanced neurodegenerative states might impact the sensitivity of neuroimaging data and limit the interpretability of the association with TSH levels. In contrast with previously published data, the majority of included cases in the current study had TSH levels within the clinically normal range, and the two cases with eventual overt hypothyroidism were excluded. The three cases with very low TSH levels were converted to the lowest measurable value (0.01 µIU/mL) for the analysis.

In previous publications, depending on the study design and the underlying population, both the left and right hippocampi might be affected. In the current study, the right hippocampus was statistically associated with TSH in the total population. For the left hippocampus, the stratification revealed a significant association in MCI. In both cases, lower TSH levels were associated with lower hippocampus volume. This highlights the novelty of the current study.

The underlying mechanisms in humans are unclear. However, rodent studies have shown an association between thyroid hormones and gene expression in the hippocampus [35], their impact on neurogenesis and synaptic plasticity [45, 46], as well as on defining white matter volume [47]. 

### Thyroid and entorhinal cortex

Reviewing published data yielded no study exploring the association between thyroid function and EC in human subjects, particularly in older adults.

The association between TSH and EC volumes in the total study population was statistically significant only on the left side. Moreover, stratification showed significant results only in the MCI group.

The EC, specifically the medial-lateral part, is the particular nest of grid cells coding in a complex computational architecture for spatial navigation and memory [20, 48, 49]. In addition to those highly specific space cells, microglial integrity of the right amygdala-hippocampus-EC was also associated with spatial learning in rodents [50]. The left EC, in contrast, was found to be rather associated with verbal memory [51]. 

Although the association between thyroid hormones and EC has been rarely studied, a few rodent studies are available. One mechanism explaining the association of hypothyroidism with cognitive impairment is associated with the regulation of the Calcium-dependent calmodulin kinase II (CaMKII), a molecule present in the EC and involved in learning and memory, by impairing the iodine-related phosphorylation [36]. The dysregulation of thyroid hormone signaling in the EC is also linked to the risk of amyloid-ß toxicity observed in Alzheimer’s disease [52]. Furthermore, developmental hypothyroidism in rodents is associated with an impairment of the entorhinal-dentate gyrus neural pathway [53]. 

No cases with hypothyroidism were included in the current study, highlighting the novelty of the revealed association between lower TSH levels and neurodegeneration in EC.

### Thyroid and cognition

The hippocampus and EC are critical brain structures that play a pivotal role in encoding and consolidating different areas of memory and learning. According to Braak staging, they are the first structures affected by tau and amyloid deposition and, consequently, by neurodegeneration during the aging process, specifically during Alzheimer’s dementia [21]. 

In a large population study, higher TSH levels in older adults were associated with lower dementia risks and better global cognitive performance [54]. On the other side, thyrotoxicosis, a severe form of hyperthyroidism, is associated with a higher risk of cognitive adversities in older adults [13]. Hyperthyroidism-associated cognitive decline might, however, be a reversible condition and needs, therefore, to be recognized and treated at early stages to avoid chronic adversities [55]. 

Previous studies on the association between lower TSH levels and cognitive impairment go along with our current results and particularly support the significant positive associations between the lower TSH levels and lower EC volumes found in MCI strata [13, 14]. 

### Thyroid and amygdala

The amygdala plays a central role in emotional processing, fear learning, empathy, and memory formation [23, 56, 57]. Dysfunction in the amygdalae is particularly associated with psychiatric disorders such as stress and affective disorders [58–61]. 

In the current study, cases with normal and pathological total scores of GDS were included. The GDS was thus adjusted for in the regression models. Similarly, both cases with and without anxiety, as perceived and reported by their study partner, were eligible. It is important to mention that in the previously cited study on premenopausal women with Graves’ disease, no significant association between the level of anxiety and the volumes of the (right and left) amygdalae was observed [34]. 

Although the current study found no association between the amygdala and TSH in middle-aged and older adults, some published studies on different populations are in favor of associations between other biomarkers of thyroid function and amygdala volumes. In the study on premenopausal women with Graves’ disease, amygdala volumes were significantly lower in affected women than in the control group (-10.4% for the left and − 13.3% for the right amygdala, both *p*_*t−test*_<0.001). After treatment, both increased significantly in size (+ 6.7% for the left and + 11.1% for the right amygdala, both *p*_*t−test*_<0.001) [34]. Similarly to hippocampus volumes, no significant correlation was found between TSH and amygdala volumes. However, the only significant (negative) correlations in this population were observed between TSH receptor antibodies (TRAb) levels, on one side, and both amygdala volumes and the right hippocampus, on the other side [34]. In a further study, the administration of Levothyroxine (L-T_4_) to patients with bipolar disorder during a depressive episode was significantly associated with decreased metabolic activity in the right amygdala and hippocampus [62].

### Strengths

The association between lower TSH levels and neurodegeneration in the hippocampus and EC structures, particularly on the left side, presents the major novelty of the study. The high number of included participants, the cognitive status-related stratification, and the bilateral volumetric analyses are major strengths. This is the first study exploring the association between TSH and hippocampus, EC, and amygdala volumes in 50-year-old and older adults. The association related to these brain structures was of certain complexity since it showed an interplay between neurodegenerative states, cognition, and the explored side of the brain. These particularities were detailed in the current analyses and opened the doors to further investigations aiming at a better understanding of the underlying mechanisms.

### Limitations

The first limitation of the study is associated with the lack of free triiodothyronine (FT3) and free Thyroxine (FT4) measurements at baseline. While both hormones reflect peripheral thyroid function, the study’s main exposure was TSH as a biomarker for central thyroid function. The study included cases without extremely pathological TSH levels to compensate for this limitation. Furthermore, TSH is negatively associated with FT_3_ and FT_4_ values and reflects, therefore, their variations even within normal ranges. The second limitation is associated with the relatively high number of participants without data on VOI, which had to be excluded from the current analyses. Technical challenges and anatomical variations during neurodegeneration might explain this deficit. To reduce this bias, cases with dementia were excluded. The third limitation is associated with the study design, seeing that cross-sectional analyses do not allow drawing a causal inference from the observed associations. This study was meant to pave the path for further structure-specific longitudinal studies.

## Conclusions

The significant association between central thyroid function and brain structures in 50-year-old and older adults highlights the importance of the body-brain interaction during aging and the role of TSH in understanding the underlying process of cognition and emotions. The bilateral volumetric analysis showed lateralization in the association between the central thyroid function and different structures of MTL, as lower TSH levels were only associated with lower hippocampus and EC volume, particularly on the left side. The degenerative remodeling observed in MCI, and later dementia, might predispose to higher affinities between brain structures and TSH. However, no causal relationship can be concluded from these cross-sectional results, and larger longitudinal studies are needed. Particularly in older adults, thyroid-brain interaction might be of complexity requiring multidisciplinary approaches, different strategies, and stratified analyses. The restrictive strategy based on excluding cases with probable hypothyroidism helped reveal a novel association between lower TSH levels and neurodegeneration in the medial temporal lobe.

## Electronic supplementary material

Below is the link to the electronic supplementary material.

Supplementary Material 1: Supplementary table 1: Adjusted models of the regression analyses with *left and right hippocampus volume* (mm^3^) as the predicted variable and TSH (µIU/mL) as the predicting variable

Supplementary Material 2: Supplementary table 2: Adjusted models of the regression analyses with *left and right entorhinal cortex volume* (mm^3^) as the predicted variable and TSH (µIU/mL) as the predicting variable

Supplementary Material 3: Supplementary table 3: Crude and adjusted models of the regression analyses with *left and right amygdala volume* (mm^3^) as the predicted variable and TSH (µIU/mL) as the predicting variable

## Data Availability

The datasets supporting the conclusions of this article are available at [http://adni.loni.usc.edu] (http://adni.loni.usc.edu) .
