# Supplementary material for: Association of hippocampus, entorhinal cortex, and amygdala with thyroid function: a bilateral volumetric analysis
Source: Thyroid Res. 2026 Feb 23;19:8. doi: 10.1186/s13044-026-00289-4 (PMC12927246; doi:10.1186/s13044-026-00289-4)
Supplement: Supplementary file 1 — Supplementary Material 1: Supplementary table 1: Adjusted models of the regression analyses with left and right hippocampus volume (mm3) as the predicted variable and TSH (µIU/mL) as the predicting variable [file 13044_2026_289_MOESM1_ESM.docx]

**Supplementary Table 1:** Associations between right and left Hippocampus volume (mm^3^) and thyroid-stimulating hormone levels (µIU/mL) in the total study population, healthy controls, and mild cognitive impairment.

|  | **Left** | | | | | | **Right** | | | | | |
| --- | --- | --- | --- | --- | --- | --- | --- | --- | --- | --- | --- | --- |
| **Predictors** | **Total study participants** | | **Healthy controls** | | **Mild cognitive impairment** | | **Total study participants** | | **Healthy controls** | | **Mild cognitive impairment** | |
|  | ***ß*** ***(95% CI)*** | ***p-value*** | ***ß*** ***(95% CI)*** | ***p-value*** | ***ß*** ***(95% CI)*** | ***p-value*** | ***ß*** ***(95% CI)*** | ***p-value*** | ***ß*** ***(95% CI)*** | ***p-value*** | ***ß (95% CI)*** | ***p-value*** |
| TSH (µIU/mL) | 25.00 (-0.02, 51.00) | 0.050 | 0.18 (-33.00, 33.00) | >0.900 | 44.00 (7.50, 81.00) | **0.018** | 29.00 (4.00, 54.00) | **0.023** | 22.00 (-12.00, 55.00) | 0.200 | 35.00 (-1.90, 71.00) | 0.063 |
| Age (years) | -26.00 (-30.00, -21.00) | **<0.001** | -23.00 (-29.00, -17.00) | **<0.001** | -28.00 (-33.00, -22.00) | **<0.001** | -29.00 (-33.00, -25.00) | **<0.001** | -28.00 (-34.00, -21.00) | **<0.001** | -30.00 (-35.00, -25.00) | **<0.001** |
| Sex |  |  |  |  |  |  |  |  |  |  |  |  |
| Female | — |  | — |  | — |  | — |  | — |  | — |  |
| Male | 141.00 (74.00, 207.00) | **<0.001** | 51.00 (-45.00, 146.00) | 0.300 | 162.00 (70.00, 254.00) | **<0.001** | 138.00 (72.00, 204.00) | **<0.001** | 15.00 (-82.00, 112.00) | 0.800 | 185.00 (94.00, 276.00) | **<0.001** |
| Main diagnosis |  |  |  |  |  |  |  |  |  |  |  |  |
| Healthy controls | — |  |  |  |  |  | — |  |  |  |  |  |
| MCI | -115.00 (-182.00, -48.00) | **<0.001** |  |  |  |  | -109.00 (-175.00, -42.00) | **0.001** |  |  |  |  |
| Ethnicity |  |  |  |  |  |  |  |  |  |  |  |  |
| White | — |  | -2.80 (-141.00, 136.00) | >0.900 | -12.00 (-212.00, 188.00) | >0.900 | — |  | 61.00 (-80.00, 202.00) | 0.400 | 34.00 (-164.00, 232.00) | 0.700 |
| Black | 15.00 (-105.00, 136.00) | 0.800 | — |  | — |  | -43.00 (-163.00, 78.00) | 0.500 | — |  | — |  |
| Other | 15.00 (-118.00, 148.00) | 0.800 | 26.00 (-186.00, 238.00) | 0.800 | 13.00 (-261.00, 288.00) | >0.900 | 55.00 (-78.00, 187.00) | 0.400 | 156.00 (-59.00, 372.00) | 0.200 | 61.00 (-212.00, 334.00) | 0.700 |
| Education (years) | -9.70 (-21.00, 1.20) | 0.082 | -20.00 (-35.00, -5.10) | **0.009** | -1.00 (-16.00, 14.00) | 0.900 | -15.00 (-26.00, -4.00) | **0.007** | -24.00 (-39.00, -8.70) | **0.002** | -6.90 (-22.00, 8.30) | 0.400 |
| APOE ε4 status | -41.00 (-84.00, 2.90) | 0.067 | -26.00 (-90.00, 38.00) | 0.400 | -37.00 (-96.00, 22.00) | 0.200 | -46.00 (-89.00, -2.60) | **0.038** | -44.00 (-109.00, 21.00) | 0.200 | -37.00 (-96.00, 21.00) | 0.200 |
| ADAS_13_ total score (points) | -25.00 (-30.00, -20.00) | **<0.001** | -12.00 (-21.00, -3.20) | **0.008** | -28.00 (-34.00, -22.00) | **<0.001** | -23.00 (-28.00, -18.00) | **<0.001** | -11.00 (-20.00, -2.20) | **0.015** | -26.00 (-32.00, -20.00) | **<0.001** |
| GDS total score (points) | -4.80 (-25.00, 16.00) | 0.600 | -12.00 (-45.00, 22.00) | 0.500 | -2.10 (-28.00, 24.00) | 0.900 | -5.80 (-26.00, 15.00) | 0.600 | -25.00 (-59.00, 9.60) | 0.200 | 1.80 (-24.00, 28.00) | 0.900 |
| BMI | 10.00 (5.00, 16.00) | **<0.001** | 8.10 (0.97, 15.00) | **0.026** | 11.00 (3.40, 19.00) | **0.005** | 12.00 (6.40, 17.00) | **<0.001** | 7.40 (0.17, 15.00) | **0.045** | 14.00 (6.60, 22.00) | **<0.001** |
| ICV (mm^3^) | 0.00 (0.00, 0.00) | **<0.001** | 0.00 (0.00, 0.00) | **<0.001** | 0.00 (0.00, 0.00) | **0.001** | 0.00 (0.00, 0.00) | **<0.001** | 0.00 (0.00, 0.00) | **<0.001** | 0.00 (0.00, 0.00) | **<0.001** |
| MRI-Scanner |  |  |  |  |  |  |  |  |  |  |  |  |
| 3 Tesla | — |  | — |  | — |  | — |  | — |  | — |  |
| Accelerated 1 Tesla | -5.90 (-99.00, 88.00) | >0.900 | 45.00 (-106.00, 197.00) | 0.600 | -70.00 (-199.00, 59.00) | 0.300 | -33.00 (-126.00, 61.00) | 0.500 | -2.20 (-157.00, 152.00) | >0.900 | -77.00 (-205.00, 51.00) | 0.200 |
| Non-Accelerated 1 Tesla | -62.00 (-123.00, -2.30) | **0.042** | -20.00 (-94.00, 54.00) | 0.600 | -117.00 (-213.00, -21.00) | **0.017** | -111.00 (-171.00, -51.00) | **<0.001** | -66.00 (-142.00, 9.20) | 0.085 | -154.00 (-250.00, -59.00) | **0.002** |
| Period between TSH and MRI (days) | -0.06 (-0.40, 0.28) | 0.700 | -0.04 (-0.34, 0.26) | 0.800 | -1.30 (-4.10, 1.50) | 0.400 | -0.06 (-0.40, 0.27) | 0.700 | -0.02 (-0.32, 0.28) | 0.900 | -1.90 (-4.70, 0.83) | 0.200 |
| **ADAS_13_:** Alzheimer’s Disease Assessment Scale – 13 items, **APOE:** Apolipoprotein E, **CI:** Confidence Interval, **ICV:** Intracranial Volume, **MCI:** Mild Cognitive Impairment, **MRI:** Magnetic Resonance Imaging, **TSH:** Thyroid Stimulating Hormone. | | | | | | | | | | | | |
