# Supplementary material for: Association of hippocampus, entorhinal cortex, and amygdala with thyroid function: a bilateral volumetric analysis
Source: Thyroid Res. 2026 Feb 23;19:8. doi: 10.1186/s13044-026-00289-4 (PMC12927246; doi:10.1186/s13044-026-00289-4)
Supplement: Supplementary file 2 — Supplementary Material 2: Supplementary table 2: Adjusted models of the regression analyses with left and right entorhinal cortex volume (mm3) as the predicted variable and TSH (µIU/mL) as the predicting variable [file 13044_2026_289_MOESM2_ESM.docx]

**Supplementary Table 2:** Associations between right and left entorhinal Cortex volume (mm^3^) and thyroid-stimulating hormone levels (µIU/mL) in the total study population, healthy controls, and mild cognitive impairment.

|  | **Left** | | | | | | **Right** | | | | | |
| --- | --- | --- | --- | --- | --- | --- | --- | --- | --- | --- | --- | --- |
| **Predictors** | **Total study participants** | | **Healthy controls** | | **Mild cognitive impairment** | | **Total study participants** | | **Healthy controls** | | **Mild cognitive impairment** | |
|  | ***ß (95% CI)*** | ***p-value*** | ***ß (95% CI)*** | ***p-value*** | ***ß (95% CI)*** | ***p-value*** | ***ß (95% CI)*** | ***p-value*** | ***ß (95% CI)*** | ***p-value*** | ***ß*** ***(95% CI)*** | ***p-value*** |
| TSH (µIU/mL) | 31.00 (9.40, 52.00) | **0.005** | 5.80 (-23.00, 35.00) | 0.700 | 54.00 (24.00, 84.00) | **<0.001** | 13.00 (-10.00, 35.00) | 0.300 | 18.00 (-15.00, 50.00) | 0.300 | 9.00 (-23.00, 41.00) | 0.600 |
| Age (years) | -7.60 (-11.00, -4.20) | **<0.001** | -9.40 (-15.00, -4.10) | **<0.001** | -6.70 (-11.00, -2.30) | **0.003** | -7.90 (-12.00, -4.30) | **<0.001** | -8.80 (-15.00, -2.90) | **0.004** | -7.30 (-12.00, -2.60) | **0.002** |
| Sex |  |  |  |  |  |  |  |  |  |  |  |  |
| Female | — |  | — |  | — |  | — |  | — |  | — |  |
| Male | 129.00 (73.00, 185.00) | **<0.001** | 134.00 (49.00, 219.00) | **0.002** | 107.00 (31.00, 182.00) | **0.006** | 110.00 (50.00, 169.00) | **<0.001** | 118.00 (24.00, 213.00) | **0.015** | 83.00 (3.90, 162.00) | **0.040** |
| Main diagnosis |  |  |  |  |  |  |  |  |  |  |  |  |
| Healthy controls | — |  |  |  |  |  | — |  |  |  |  |  |
| MCI | -53.00 (-110.00, 3.10) | 0.064 |  |  |  |  | -75.00 (-135.00, -15.00) | **0.015** |  |  |  |  |
| Ethnicity |  |  |  |  |  |  |  |  |  |  |  |  |
| White | — |  | 179.00 (56.00, 302.00) | **0.005** | 33.00 (-130.00, 197.00) | 0.700 | — |  | 35.00 (-102.00, 172.00) | 0.600 | -74.00 (-246.00, 97.00) | 0.400 |
| Black | -113.00 (-215.00, -11.00) | **0.030** | — |  | — |  | 16.00 (-92.00, 124.00) | 0.800 | — |  | — |  |
| Other | -27.00 (-139.00, 85.00) | 0.600 | 107.00 (-82.00, 295.00) | 0.300 | 80.00 (-145.00, 306.00) | 0.500 | 27.00 (-93.00, 146.00) | 0.700 | 189.00 (-21.00, 399.00) | 0.078 | -156.00 (-392.00, 80.00) | 0.200 |
| Education (years) | -1.10 (-10.00, 8.10) | 0.800 | -8.70 (-22.00, 4.60) | 0.200 | 3.70 (-8.90, 16.00) | 0.600 | -4.00 (-14.00, 5.80) | 0.400 | -3.30 (-18.00, 12.00) | 0.700 | -4.70 (-18.00, 8.50) | 0.500 |
| APOE ε4 status | -23.00 (-60.00, 14.00) | 0.200 | -21.00 (-78.00, 36.00) | 0.500 | -13.00 (-61.00, 36.00) | 0.600 | -2.10 (-41.00, 37.00) | >0.900 | -1.00 (-64.00, 62.00) | >0.900 | 9.30 (-41.00, 60.00) | 0.700 |
| ADAS_13_ total score (points) | -13.00 (-17.00, -8.50) | **<0.001** | -0.29 (-8.20, 7.60) | >0.9 | -17.00 (-22.00, -12.00) | **<0.001** | -12.00 (-16.00, -7.60) | **<0.001** | -2.10 (-11.00, 6.80) | 0.600 | -16.00 (-21.00, -10.00) | **<0.001** |
| GDS total score (points) | -4.80 (-22.00, 12.00) | 0.600 | -16.00 (-46.00, 14.00) | 0.300 | 0.94 (-21.00, 22.00) | >0.900 | -0.14 (-18.00, 18.00) | >0.900 | -16.00 (-50.00, 17.00) | 0.300 | 5.90 (-17.00, 28.00) | 0.600 |
| BMI | 0.61 (-3.90, 5.10) | 0.800 | 2.60 (-3.70, 9.00) | 0.400 | -2.60 (-9.00, 3.70) | 0.400 | 3.60 (-1.20, 8.30) | 0.140 | 5.70 (-1.30, 13.00) | 0.110 | 1.90 (-4.70, 8.50) | 0.600 |
| ICV (mm^3^) | 0.00 (0.00, 0.00) | **<0.001** | 0.00 (0.00, 0.00) | **<0.001** | 0.00 (0.00, 0.00) | **<0.001** | 0.00 (0.00, 0.00) | **<0.001** | 0.00 (0.00, 0.00) | **<0.001** | 0.00 (0.00, 0.00) | **<0.001** |
| MRI-Scanner |  |  |  |  |  |  |  |  |  |  |  |  |
| 3 Tesla | — |  | — |  | — |  | — |  | — |  | — |  |
| Accelerated 1 Tesla | -101.00 (-180.00, -22.00) | **0.012** | -25.00 (-160.00, 109.00) | 0.700 | -133.00 (-239.00, -27.00) | **0.014** | -223.00 (-307.00, -139.00) | **<0.001** | -237.00 (-387.00, -86.00) | **0.002** | -235.00 (-346.00, -124.0) | **<0.001** |
| Non-Accelerated 1 Tesla | -132.00 (-183.00, -82.00) | **<0.001** | -153.00 (-219.00, -87.00) | **<0.001** | -121.00 (-200.00, -42.00) | **0.003** | -253.00 (-306.00, -199.00) | **<0.001** | -257.00 (-330.00, -183.00) | **<0.001** | -256.00 (-338.00, -173.0) | **<0.001** |
| Period between TSH and MRI (days) | 0.06 (-0.23, 0.34) | 0.700 | 0.01 (-0.26, 0.27) | >0.900 | 0.80 (-1.50, 3.10) | 0.500 | -0.09 (-0.40, 0.21) | 0.600 | -0.12 (-0.41, 0.18) | 0.400 | 1.10 (-1.30, 3.50) | 0.400 |
| **ADAS_13_:** Alzheimer’s Disease Assessment Scale – 13 items, **APOE:** Apolipoprotein, **CI:** Confidence Interval, **ICV:** Intracranial Volume, **MCI:** Mild Cognitive Impairment, **MRI:** Magnetic Resonance Imaging, **TSH:** Thyroid Stimulating Hormone. | | | | | | | | | | | | |
