# Supplementary material for: Association of hippocampus, entorhinal cortex, and amygdala with thyroid function: a bilateral volumetric analysis
Source: Thyroid Res. 2026 Feb 23;19:8. doi: 10.1186/s13044-026-00289-4 (PMC12927246; doi:10.1186/s13044-026-00289-4)
Supplement: Supplementary file 3 — Supplementary Material 3: Supplementary table 3: Crude and adjusted models of the regression analyses with left and right amygdala volume (mm3) as the predicted variable and TSH (µIU/mL) as the predicting variable [file 13044_2026_289_MOESM3_ESM.docx]

**Supplementary Table 3:** Associations between right and left amygdala (mm^3^) and thyroid-stimulating hormone levels (µIU/mL) in the total study population, healthy controls, and mild cognitive impairment.

|  | **Left** | | | | | | **Right** | | | | | |
| --- | --- | --- | --- | --- | --- | --- | --- | --- | --- | --- | --- | --- |
| **Predictors** | **Total study participants** | | **Healthy controls** | | **Mild cognitive impairment** | | **Total study participants** | | **Healthy controls** | | **Mild cognitive impairment** | |
|  | ***ß*** ***(95% CI)*** | ***p-value*** | ***ß*** ***(95% CI)*** | ***p-value*** | ***ß*** ***(95% CI)*** | ***p-value*** | ***ß*** ***(95% CI)*** | ***p-value*** | ***ß*** ***(95% CI)*** | ***p-value*** | ***ß*** ***(95% CI)*** | ***p-value*** |
| TSH (µIU/mL) | 8.50 (-4.00, 21.00) | 0.2 | 5.70 (-12.00, 23.00) | 0.500 | 12.00 (-5.60, 30.00) | 0.200 | 5.80 (-6.50, 18.00) | 0.400 | 6.30 (-10.00, 23.00) | 0.500 | 6.40 (-12.00, 24.00) | 0.500 |
| Age (years) | -11.00 (-13.00, -9.10) | **<0.001** | -10.00 (-13.00, -6.90) | **<0.001** | -12.00 (-14.00, -9.20) | **<0.001** | -10.00 (-12.00, -8.00) | **<0.001** | -7.80 (-11.00, -4.80) | **<0.001** | -11.00 (-14.00, -8.60) | **<0.001** |
| Sex |  |  |  |  |  |  |  |  |  |  |  |  |
| Female | — |  |  |  |  |  | — |  |  |  |  |  |
| Male | -42.00 (-75.00, -8.80) | **0.013** |  |  |  |  | -44.00 (-77.00, -11.00) | **0.009** |  |  |  |  |
| Main diagnosis |  |  |  |  |  |  |  |  |  |  |  |  |
| Healthy controls | — |  | — |  | — |  | — |  | — |  | — |  |
| MCI | 89.00 (56.00, 121.00) | **<0.001** | 33.00 (-18.00, 85.00) | 0.200 | 111.00 (67.00, 155.00) | **<0.001** | 124.00 (92.00, 157.00) | **<0.001** | 72.00 (24.00, 121.00) | **0.003** | 147.00 (103.00, 192.00) | **<0.001** |
| Ethnicity |  |  |  |  |  |  |  |  |  |  |  |  |
| White | — |  | 14.00 (-61.00, 88.00) | 0.700 | -2.70 (-98.00, 93.00) | >0.900 | — |  | 26.00 (-44.00, 96.00) | 0.500 | -42.00 (-140.00, 55.00) | 0.400 |
| Black | -6.50 (-66.00, 53.00) | 0.8 | — |  | — |  | 1.80 (-57.00, 61.00) | >0.900 | — |  | — |  |
| Other | 0.18 (-66.00, 66.00) | >0.9 | 63.00 (-51.00, 177.00) | 0.300 | -35.00 (-166.00, 97.00) | 0.600 | -0.75 (-66.00, 64.00) | >0.900 | 59.00 (-48.00, 166.00) | 0.300 | -57.00 (-191.00, 76.00) | 0.400 |
| Education (years) | -1.00 (-6.40, 4.40) | 0.7 | -0.90 (-9.00, 7.20) | 0.800 | -1.10 (-8.40, 6.30) | 0.800 | -5.00 (-10.00, 0.37) | 0.068 | -6.90 (-15.00, 0.71) | 0.075 | -4.30 (-12.00, 3.20) | 0.300 |
| APOE ε4 status | -12.00 (-34.00, 9.40) | 0.3 | -10.00 (-45.00, 24.00) | 0.600 | -7.90 (-36.00, 20.00) | 0.600 | -8.70 (-30.00, 13.00) | 0.400 | -16.00 (-48.00, 17.00) | 0.300 | -1.40 (-30.00, 27.00) | >0.900 |
| ADAS_13_ total score (points) | -9.40 (-12.00, -7.00) | **<0.001** | -2.70 (-7.50, 2.10) | 0.300 | -11.00 (-14.00, -8.50) | **<0.001** | -8.80 (-11.00, -6.40) | **<0.001** | -3.80 (-8.30, 0.72) | 0.100 | -10.00 (-13.00, -7.50) | **<0.001** |
| GDS total score (points) | -6.30 (-16.00, 3.90) | 0.2 | -14.00 (-32.00, 3.60) | 0.120 | -2.20 (-15.00, 10.00) | 0.700 | -3.50 (-14.00, 6.50) | 0.500 | -12.00 (-29.00, 4.80) | 0.200 | -0.07 (-13.00, 13.00) | >0.900 |
| BMI | 3.10 (0.47, 5.70) | **0.021** | 3.00 (-0.77, 6.90) | 0.120 | 2.70 (-1.00, 6.40) | 0.200 | 3.90 (1.40, 6.50) | **0.003** | 4.60 (1.00, 8.20) | **0.012** | 3.10 (-0.61, 6.90) | 0.100 |
| ICV (mm^3^) | 0.00 (0.00, 0.00) | **<0.001** | 0.00 (0.00, 0.00) | **<0.001** | 0.00 (0.00, 0.00) | **<0.001** | 0.00 (0.00, 0.00) | **<0.001** | 0.00 (0.00, 0.00) | **<0.001** | 0.00 (0.00, 0.00) | **<0.001** |
| MRI-Scanner |  |  |  |  |  |  |  |  |  |  |  |  |
| 3 Tesla | — |  | — |  | — |  | — |  | — |  | — |  |
| Accelerated 1 Tesla | -85.00 (-132.00, -39.00) | **<0.001** | -76.00 (-157.00, 5.50) | 0.068 | -93.00 (-155.00, -31.00) | **0.003** | -138.00 (-184.00, -92.00) | **<0.001** | -117.00 (-193.00, -40.00) | **0.003** | -147.00 (-209.00, -84.00) | **<0.001** |
| Non-Accelerated 1 Tesla | -86.00 (-116.00, -56.00) | **<0.001** | -94.00 (-133.00, -54.00) | **<0.001** | -80.00 (-126.00, -34.00) | **<0.001** | -181.00 (-210.00, -152.00) | **<0.001** | -190.00 (-228.00, -153.00) | **<0.001** | -174 (-221.00, -128.00) | **<0.001** |
| Period between TSH and MRI (days) | 0.04 (-0.13, 0.20) | 0.7 | 0.03 (-0.13, 0.19) | 0.700 | 0.33 (-1.00, 1.70) | 0.600 | -0.02 (-0.19, 0.14) | 0.800 | -0.02 (-0.17, 0.13) | 0.800 | -0.39 (-1.70, 0.98) | 0.600 |
| **ADAS_13_:** Alzheimer’s Disease Assessment Scale – 13 items, **APOE:** Apolipoprotein, **CI:** Confidence Interval, **ICV:** Intracranial Volume, **MCI:** Mild Cognitive Impairment, **MRI:** Magnetic Resonance Imaging, **TSH:** Thyroid Stimulating Hormone. | | | | | | | | | | | | |
